# ﻿The complete mitogenome of the potentially invasive flatworm *Australopacificaatrata* (Platyhelminthes, Geoplanidae) displays unusual features common to other Rhynchodeminae

**DOI:** 10.3897/zookeys.1110.83228

**Published:** 2022-07-05

**Authors:** Romain Gastineau, Leigh Winsor, Jean-Lou Justine

**Affiliations:** 1 Institute of Marine and Environmental Sciences, University of Szczecin, Szczecin, Poland University of Szczecin Szczecin Poland; 2 College of Science and Engineering, James Cook University, Townsville, Queensland, Australia James Cook University Condon Australia; 3 ISYEB, Institut de Systématique, Évolution, Biodiversité (UMR7205 CNRS, EPHE, MNHN, UPMC, Université des Antilles), Muséum National d’Histoire Naturelle, CP 51, 55 rue Buffon, 75231 Paris Cedex 05, France Université des Antilles Paris France

**Keywords:** Biomonitoring, Continenticola, *cox2* gene, multigene phylogeny, next generation sequencing, *Parakontikiaventrolineata*, Tricladida

## Abstract

We sequenced the complete mitochondrial genome of the flatworm *Australopacificaatrata*. The species, originally described from New South Wales, Australia, has been found in various locations in the British Isles, New Zealand and in the United States of America; it is thus potentially invasive. The genome is 16513 bp long, encodes for 12 protein coding genes, two ribosomal RNA genes and 20 tRNA genes, and is completely colinear with the other two available Rhynchodeminae. In addition, it shares with them some unusual characters discriminating them from members of the other subfamilies of Geoplanidae, the most noticeable being the extra length of its *cox2* gene. The data allow a reliable multigene phylogeny to be derived, and also provide a means of accurate biomonitoring of possible invasiveness by *A.atrata*.

## ﻿Introduction

In the recent years, an increasing number of reports have emerged from Europe and abroad concerning invasive terrestrial flatworms of the family Geoplanidae Stimpson, 1857 (Winsor et al. 2004; Sluys 2016). The most recent classification of the Geoplanidae (Almeida et al. 2021) lists five subfamilies within the Geoplanidae, among which three might be of particular concern in the context of biological invasions. The most infamous species in these subfamilies are probably the ‘hammerhead flatworm’ *Bipaliumkewense* Moseley, 1878 (Bipaliinae Von Graff, 1896) from South East Asia (Winsor 1983; Justine et al. 2018), followed by *Obamanungara* Carbayo, Álvarez-Presas, Jones & Riutort, 2016 (Geoplaninae Stimpson, 1857) from South America (Carbayo et al. 2016; Lago-Barcia et al. 2019; Justine et al. 2020b; Fourcade 2021) and *Platydemusmanokwari* de Beauchamp, 1963 (Rhynchodeminae Von Graff, 1896) from Papua New Guinea (Justine et al. 2014; Justine et al. 2015; Gastineau et al. 2020; Justine et al. 2021). With their large size and their feeding habits, these predators of soil invertebrates have attracted most attention.

**Figure 1.**
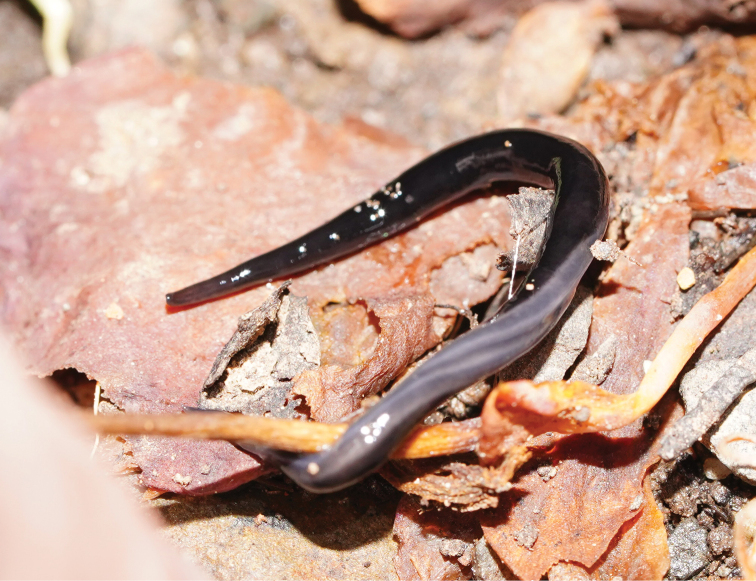
Living flatworm *Australopacificaatrata* (Steel, 1897), showing underside of mid body.

**Figure 2.**
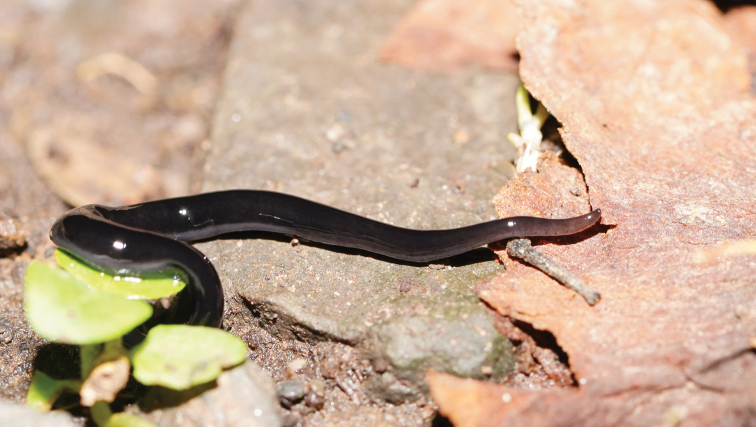
Living flatworm *Australopacificaatrata* (Steel, 1897), dorsal view.

Two species, smaller than the aforementioned large ones and with a mostly scavenging behaviour, are also potentially invasive (Winsor et al. 2004). The first is *Parakontikiaventrolineata* (Dendy, 1892) Winsor 1991; the species is about 30 mm in length with a dark grey-black body, with longitudinal black stripes on the dorsum, and with the eponymous paired greyish stripes on its ventral surface. Originally from Queensland, Australia, it has been found elsewhere in Australia, and has also been reported in France (Justine et al. 2014; Gastineau et al. 2020), Spain (Álvarez-Presas et al. 2014) and possibly in South Africa (Jones et al. 1998). Also New Zealand, Hawaii, United Kingdom, Madeira, USA, and Mexico (records summarized in Winsor et al. 2004), Costa-Rica, Colombia, Ecuador, Chile, Argentina, and confirmed for South Africa (iNaturalist records, https://www.inaturalist.org/). The second species is *Australopacificaatrata* (Steel, 1897). Originally described as *Geoplanaatrata* from New South Wales, Australia, it was transferred to *Parakontikia* on the basis of its external morphology and internal anatomy (Winsor 1991), and subsequently to *Australopacifica* (Jones, 2019). The formal definition of *Australopacifica* is “Geoplanidae, but not classifiable into the present taxonomic genera because of insufficient morphological information; geographical distribution largely in Australasia and Indo Pacific islands. A collective group to temporarily assign *species inquirendae* and *nomina dubia*” (Ogren and Kawakatsu 1991). It is also found in other Australian states and territories: Victoria (Winsor 1973), Queensland (Winsor 1997), the Australian Capital Territory, and Tasmania (Atlas of Living Australia records, https://www.ala.org.au/). The species has been recorded in New Zealand (Winsor et al. 2004), in Wales and South England (Jones 2019), and in California, USA (iNaturalist records). The species superficially resembles *Pa.ventrolineata*, except that it has a uniformly black dorsum, with a characteristic median dark stripe on its ventral surface (Figs 1, 2). Both species are gregarious, collectively predating upon slugs and snails and other soil species such as isopods (Smith 1979; Barker 1989; Winsor et al. 2004), and are also necrophagous scavengers (Smith 1979; Jones 2019). They have been recorded in France and the UK on various fallen fruit lying on the soil, and in cavities in growing strawberries; whether the planarians are simply attracted to rotting fruit or actually feeding on the fruit remains has yet to be adequately explained (Jones 2019; Justine et al. 2020b).

In recent years, several complete mitochondrial genomes from different species of terrestrial invasive flatworms have been sequenced (Solà et al. 2015; Gastineau et al. 2019; Gastineau et al. 2020; Gastineau and Justine, 2020; Justine et al. 2020a; Justine et al. 2022). Full mitogenomes provide a complete sequence for the widely used molecular barcode COI or *cox1* (the gene of the cytochrome c oxidase subunit 1) and allows us to perform robust multigene phylogenies. Full mitogenomes may provide additional information such as those related to the gene order or the presence of pseudogenes resulting from duplications. It also prevents amplification of nuclear copies of mitochondrial pseudogenes by PCR, a problem that sometimes occurs in molecular barcoding (Song et al. 2008). In the case of terrestrial flatworms, earlier reports have noted that there are mitogenomic features common to several species of the same subfamily that are not present in other subfamilies (Solà et al. 2015; Gastineau et al. 2020; Gastineau and Justine 2020; Justine et al. 2020a). However, the number of available mitogenomes remains still too low to allow for more general conclusions. Prior to this study, GenBank did not contain any sequence corresponding to the genus *Australopacifica*.

Here we report the complete mitochondrial genome of a morphologically identified specimen of *A.atrata* and compare it with other species. We provide further evidence concerning the specific features of the mitogenomes of Rhynchodeminae compared to land flatworms in other subfamilies. We present a multigene phylogeny demonstrating its proximity with *Pa.ventrolineata*. Finally, we discuss the next steps that should take place in the investigation of invasive terrestrial flatworms by means of next generation sequencing.

## ﻿Material and methods

### ﻿Biological material

All specimens were collected from a native plant nursery in Maffra, Victoria, Australia (37°57'S, 146°59'E), from November 2019 to February 2020 and identified on the basis of external morphology and colour pattern (Steel 1897; Winsor 1973). They were killed in boiling water and preserved in 95% ethanol. Specimens were registered in the collection of the Muséum National d’Histoire Naturelle (MNHN; Paris, France) and in the collection of one of us (LW field number, as MNHN JL368 (2 specimens LW1874), JL372 (3 specimens LW1880) and JL374 (3 specimens LW1883). One specimen MNHN JL374 was destroyed for the molecular analysis.

### ﻿Sequencing, assembly and annotation

A tissue sample in 95% ethanol was sent to the Beijing Genomics Institute (BGI) in Shenzhen, China, where DNA extraction and sequencing took place. A total of ca 40 million of 150 base pair clean paired-end reads was obtained on a DNBseq platform. The reads were assembled using SPAdes 3.14.0 (Bankevich et al. 2012), with a k-mer of 125. The annotation was made with the help of MITOS (Bernt et al. 2013) and manually curated, using Sequin 15.50 and the genetic code 9. In particular, the ribosomal genes needed to be located by alignments with reference sequences of *O.nungara* (KP208777). In such cases, alignments were performed with MEGAX (Kumar et al. 2018). The tRNA were also verified with Arwen v1.2 command line, using the -gcflatworm option (Laslett and Canbäck 2008). The map of the mitogenome was generated using OGDRAW (Lohse et al. 2013).

### ﻿Multigene phylogenies

A phylogeny was inferred with the amino-acid sequences of the conserved mitochondrial proteins, following a protocol adapted from Justine et al. (2021). Sequences were concatenated by alphabetic order, aligned using MAFFT 7 (Katoh and Standley 2013) using the -auto option and the resulting alignments was trimmed by trimAl (Capella-Gutiérrez et al. 2009), using the -automated1 option. Phylogenies were conducted using RaxML 8.0 (Stamatakis 2014), with the best tree out of 100 computed for 1000 bootstrap replicates and using the MtArt evolution model (Abascal et al. 2007) with the GAMMA model of rate heterogeneity and an estimate of proportion of invariable sites (PROTGAMMAIMTART option). *Prosthiostomumsiphunculus* Delle Chiaje (KT363736) (Polycladida) was used as an outgroup.

## ﻿Results

### ﻿Mitogenome

The mitogenome was retrieved among the other contigs from the assembly file, in the form of a contig containing all conserved coding parts, with redundant extremities which allowed us to circularize it. The genome has been deposited on GenBank with accession number OM456243.

**Figure 3.**
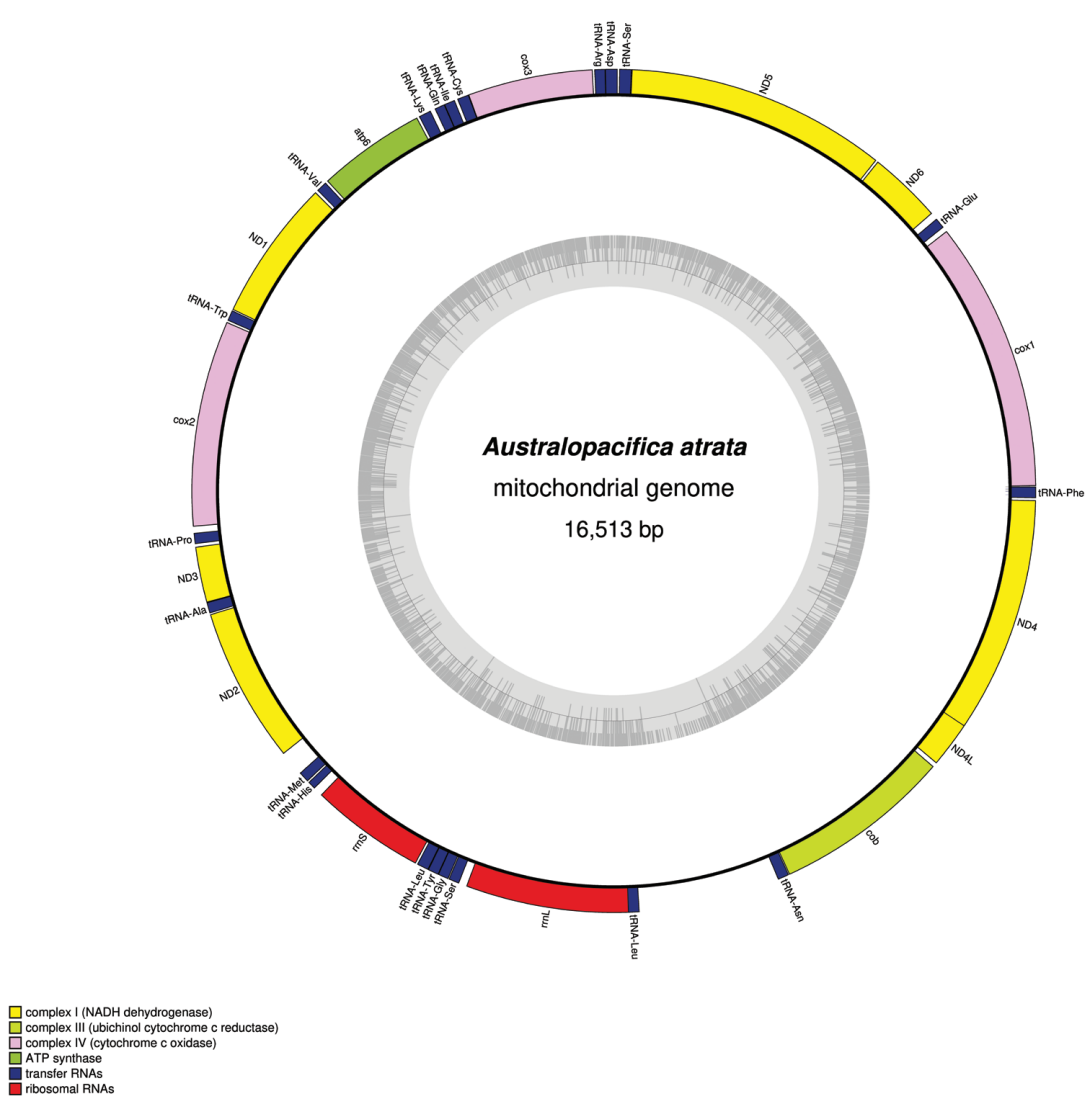
Map of the mitochondrial genome of *Australopacificaatrata* (Steel, 1897).

The mitogenome is 16513 bp long. Its composition is 4810 A, 1248 C, 2461 G and 7994 T. It encodes for 12 protein coding genes, 2 ribosomal RNA genes and 20 t RNA genes (Fig. 3). It is strictly colinear with the mitogenomes of the other Rhynchodeminae, *Pl.manokwari* and *Pa.ventrolineata*, including the t RNA. Most of the protein-coding genes start with an ATG, except *ND4*, which starts with a GTG overlapping *ND4L*, and *ND2*, for which no canonical start codon could be found. The *ND5* gene has a premature stop due to the presence of tRNA-Ser. No *tRNA-Thr* could be detected. Similar to *Pl.manokwari* and *Pa.ventrolineata*, the *cox2* gene was found to be unusually long, with an extension fragment located in the central part of the open reading frame. Table 1 lists the size of the *cox2* genes for the available mitogenomes of Continenticola (Tricladida).

### ﻿Phylogeny

**Figure 4.**
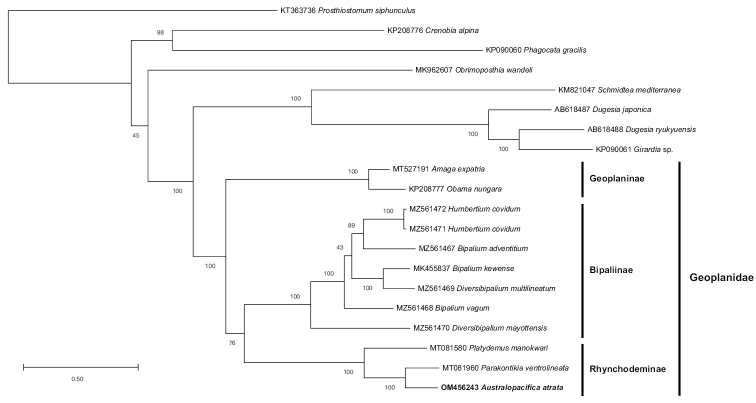
Maximum Likelihood phylogenetic tree obtained from concatenated amino-acid sequences of the mitochondrial proteins of *Australopacificaatrata* (Steel, 1897) and other flatworms. The tree with the best likelihood is shown (-75607.404300). Subfamilies of Geoplanidae are indicated on the right.

The maximum likelihood phylogenetic tree (Fig. 4) easily distinguishes the three families in the Continenticola for which mitogenomes are available, namely the Planariidae, Dugesiidae and Geoplanidae. Within the cluster of Geoplanidae, two major groups emerge: the Geoplaninae, and a cluster containing the Bipaliinae and Rhynchodeminae. The tree also retrieved *A.atrata* as the sister species of *Pa.ventrolineata* with support of 100%.

## ﻿Discussion

The mitogenome of *A.atrata* is shorter than those of the two other Rhynchodeminae*Pl.manokwari* (19959 bp, MT081580) and *Pa.ventrolineata* (17210 bp, MT081960), but larger than the largest recorded for a Bipaliinae, *Bipaliumvagum* Jones & Sterrer, 2005 (17149 bp, MZ561468) or the Geoplaninae, *O.nungara* (14909 bp, KP208777) and *Amagaexpatria* (14962 bp, MT527191). However, the presence of repeated sequences in the mitogenome of Geoplanidae has been strongly suggested (Solà et al. 2015). These repeated portions cannot be fully resolved by short reads sequencing, so the criteria of the length of the mitogenome should be taken with care.

So far, the Rhynchodeminae can be differentiated from Bipaliinae and Geoplaninae by the position of the *t RNA-Cys*. For *A.atrata*, *Pl.manokwari* and *Pa.ventrolineata*, it is located between the protein coding genes *cox3* and *atp6*, clustering with *t RNA-Ile*, *t RNA-Gln* and *t RNA-Lys*, as the first t RNA of this cluster. For *B.kewense* as well as *O.nungara* and *A.expatria*, it is located between the protein coding gene *ND2* and the 12S rRNA gene, clustering with *t RNA-Met* and *t RNA-His*, located after these two t RNA. Another difference regarding t RNA is the apparent lack of a tRNA-Thr among all Rhynchodeminae. Among the other species, *t RNA-Thr* has been found between the 16S rRNA gene and the protein-coding *cob* gene, clustering with *t RNA-Leu* and *t RNA-Asn*. It is worth emphasising here that there is a difference between the Geoplaninae, in which the order of this cluster is 16S, *t RNA-Thr*, *t RNA-Leu*, *t RNA-Asn*, *cob* and *B.kewense*, in which *t RNA-Leu* and *t RNA-Thr* exchange their positions. However, it might be noted that for the recently described species of Bipaliinae, *Humbertiumcovidum* Justine, Gastineau, Gros, Gey, Ruzzier, Charles & Winsor, 2022 (Justine et al. 2022), it was also impossible to find a tRNA-Thr. Therefore, it would be better to take a conservative view of this feature, and not to assign it a too high value for classification as a molecular synapomorphy.

Several studies failed to find a start codon for various mitochondrial genes of terrestrial flatworms such as *O.nungara*, *A.expatria*, *Diversibipaliummultilineatum* Makino & Shirasawa, 1983 and *B.vagum* (Solà et al. 2015; Justine et al. 2020a, 2022). This also seems to be the case for other members of the Continenticola, including Planariidae and Dugesiidae such as *Crenobiaalpina* Dana (KP208776) (Solà et al. 2015), *Schmidteamediterranea* Benazzi, Baguñà, Ballester, Puccinelli & Del Papa (JX398125) (Ross et al. 2016), *Dugesiaryukyuensis* Kawakatsu (AB618488), and *Dugesiajaponica* Ichikawa & Kawakatsu (AB618487) (Sakai and Sakaizumi 2012).

For all three Rhynchodeminae, the *ND5* gene has an early termination because of the presence of tRNA-Ser immediately following the last T residue of the gene, for which we suspect that the functional TAA stop codon is obtained by being completed by the addition of 3’ A residues to the mRNA, while a canonical stop codon was found for all other species. Also, it is interesting to note that there is an overlap between the *ND4L* and the *ND4* genes, and that the size of this overlap is always 32 bp. This character, which is common to *Pl.manokwari* and *Pa.ventrolineata*, has been also reported in *O.nungara* and *C.alpina*. A rapid investigation of the mitogenomes of all available Bipaliinae shows that this overlap is totally lacking. Trying to simulate this overlap by extending the open reading frame at its N terminal ending leads to a fictious polypeptide with no initial methionine but that may comprise several stop codons in its early part.

As was observed in both the other Rhynchodeminae, the *cox2* gene has an important extra length. This extra length does not result from a missing stop codon, as it is located in the middle of the gene, and not on the 3’ extremity. The size of the *cox2* putative protein of *A.atrata* is nearly identical to those of *Pa.ventrolineata* (434 and 433 amino acids respectively) (Table 1). The size is comparable to *Pl.manokwari* (452 amino acids), and far bigger than those observed among Geoplaninae and Bipaliinae, where this size ranges from 225 to 260 amino acids. It is noteworthy that *Girardia* spp. also display substantially larger *cox2* genes (KP090061, MW972220). However, an alignment performed with all the amino-acid sequences showed that this extra length is not located in the middle of the ORF, as for Rhynchodeminae, but at the C terminal ending. As verified on CDD/SPARCLE (Marchler-Bauer et al. 2017; accessed on 08/24/2021), the last conserved domain of the putative protein of *Girardia* spp. seems to be approximately at the amino-acid 227, for a total length of the predicted protein of 389 amino acids. CDD/SPARCLE positions this same conserved domain at the amino-acid 348 in *A.atrata*, for a total length of the predicted protein of 434 amino acids.

**Table 1.** Sizes in amino acids (AA) of the *cox2* proteins encoded by the available mitogenomes of Continenticola (Tricladida).

Name	Family	GenBank accession number	Size of the putative *cox*2 protein (in AA)	Specific features
*Schmidteamediterranea*	Dugesiidae	JX398125	292	Start codon not determined
*Girardia* sp.	Dugesiidae	KP090061	389	Start codon not determined
*Girardiatigrina*	Dugesiidae	MW972220	389	Start codon not determined
*Dugesiajaponica*	Dugesiidae	AB618487	227	NA
*Dugesiaryukyuensis*	Dugesiidae	AB618488	230	TAA stop codon completed by the addition of 3’ A residues to the mRNA
*Crenobiaalpina*	Planariidae	KP208776	239	NA
*Phagocatagracilis*	Planariidae	KP090060	297	NA
*Obamanungara*	Geoplanidae	KP208777	259	Start codon not determined
*Amagaexpatria*	Geoplanidae	MT527191	260	Start codon not determined
*Bipaliumkewense*	Geoplanidae	MK455837	225	NA
*Bipaliumvagum*	Geoplanidae	MZ561468	229	NA
*Bipaliumadventitium*	Geoplanidae	MZ561467	227	NA
*Diversibipaliummultilineatum*	Geoplanidae	MZ561469	228	NA
*Diversibipaliummayottensis*	Geoplanidae	MZ561470	246	NA
*Humbertiumcovidum*	Geoplanidae	MZ561471, MZ561472	248	NA
*Platydemusmanokwari*	Geoplanidae	MT081580	452	NA
*Parakontikiaventrolineata*	Geoplanidae	MT081960	433	NA
*Australopacificaatrata*	Geoplanidae	OM456243	434	NA

Following this work, we see three major paths of investigation. The first one will continue to focus on Rhynchodeminae. It will be interesting to compare all the characters considered here (colinearity, composition in t RNA, type of termination for the *ND5* gene, overlap between *ND4L* and *ND4* and of course the extra length of the *cox2* gene) with more species. Of the five tribes of Rhynchodeminae, we have now sequenced representatives of two: the Rhynchodemini (i.e. *Pl.manokwari*) and the Caenoplanini (*Pa.ventrolineata* and *A.atrata*). However, living examples of many of the other species assigned to the remaining tribes will be difficult to find considering their origin and repartition, but at least one of them, Anzoplanini*Marionfyfeaadventor* Jones & Sluys, is present in Europe (Jones and Sluys 2016). Also, *Anzoplanatrilineata* Winsor, can be found in south-eastern Australia (Winsor, 2006). Thus, we hope to obtain specimens of both species in the near future. The second would be to extend the search of shared mitogenomic characters among the subfamilies which have not been investigated until now, such as Timyminae and Microplaninae. The third and last path deals with the relationships between the genera *Parakontikia* and *Australopacifica*. Our results support the earlier provisional classification of *A.atrata* within the genus *Parakontikia* (Winsor 1991). We provide here the first sequence for a species presently assigned to the genus *Australopacifica*, which contributes towards the resolution of systematic relationships of species currently included in this heterogeneous collective genus.
